# Selection and validation of reference genes for gene expression studies in *Pseudomonas brassicacearum* GS20 using real-time quantitative reverse transcription PCR

**DOI:** 10.1371/journal.pone.0227927

**Published:** 2020-01-27

**Authors:** Bianxia Bai, Jiahong Ren, Fenling Bai, Lin Hao

**Affiliations:** 1 College of Agriculture, Shanxi Agricultural University, Jinzhong, Shanxi, China; 2 The Department of Biological Science and Technology, Changzhi University, Changzhi, Shanxi, China; 3 Ecological and Environmental Research Institute of Taihang Mountain, Changzhi, Shanxi, China; Oklahoma State University, UNITED STATES

## Abstract

*Pseudomonas brassicacearum* GS20 is an antagonistic strain of bacteria recently isolated from the rhizosphere of *Codonopsis pilosula*. No validated reference gene has been indentified from *P*. *brassicacearum* to use in the normalization of real-time quantitative reverse transcription-PCR data. Therefore, in this study, nine candidate reference genes (*recA*, *gyrA*, *rpoD*, *proC*, *gmk*, *rho*, *16S*, *ftsz*, and *secA*) were assessed at different growth phases and under various growth conditions. The expression stability of these candidate genes was evaluated using BestKeeper, NormFinder and GeNorm. In general, the results showed *rho*, *rpoD* and *gyrA* were the most suitable reference genes for *P*. *brassicacearum* GS20. The relative expression of iron-regulated gene (*fhu*) was normalized to verify the reliability of the proposed reference genes under iron-replete and iron-limited conditions. The trend in relative expression was consistent with the change in siderophore production under different iron conditions. This study presents reliable reference genes for transcriptional studies in *P*. *brassicacearum* GS20 under the chosen experimental conditions.

## Introduction

Real-time quantitative reverse transcription PCR (qRT-PCR) is a technique that is widely used to monitor changes in gene expression [1]. The method is regarded to be sensitive, specific, reproducible and accurate [2], even though factors such as the quality of extracted RNA, primer selection and the efficiency of cDNA synthesis can affect the outcome [3]. Generally, these variables are minimized by relative normalization, whereby the expression of the target gene is normalized to the expression of an internal control gene [4]. Thus, choosing an internal control gene is vital to the accurate determination of relative gene expression [5]. Housekeeping genes are usually chosen as internal controls to normalize real-time qRT-PCR data; however, recent reports have claimed that the expression of housekeeping genes may vary according to the genes, cell types and experimental conditions [6]. Confirming the stability of the control genes under experimental conditions is, therefore, critical to the accurate analysis of target gene expression.

*Pseudomonas brassicacearum* GS20 is a Gram-negative soil bacterium that we have previously isolated from the rhizosphere of *Codonopsis pilosula* (Franch.) Nannf. *C*. *pilosula* is an important Chinese medicinal plant and its dried roots are commonly used in pharmaceuticals. When cultivated successively, *C*. *pilosula* can be affected by serious soil-borne diseases caused by phytopathogenic fungi; however, we found that *P*. *brassicacearum* GS20 can antagonize these phytopathogenic fungi (unpublished). Numerous studies have verified that *Pseudomonas* species interact with fungal pathogens and inhibit their growth by producing antibiotics, secreting extracellular enzymes and competing for iron [7–11]. *Pseudomonas* species acquire iron predominantly through the production of siderophores, which are small molecules with high affinity for ferric iron [12]. Siderophores can effectively utilize limited iron in soils, thus making it unavailable to phytopathogenic fungi [13]. Using bioinformatics to analyze the whole genome sequence of *P*. *brassicacearum* GS20, we found that, as with other Gram-negative bacteria, this species contained the ferric uptake regulator (*Fur*) for the regulation of iron homeostasis.

No ideal internal control gene is known for the validation of target gene expression in *P*. *brassicacearum* GS20. In this study, nine genes, including recombinase A (*recA*), DNA gyrase A (*gyrA*), pyrroline-5-carboxylate reductase (*proC*), RNA polymerase sigma factor (*rpoD*), guanylate kinase (*gmk*), termination factor Rho (*rho*), 16S ribosomal RNA (*16S*), cell division protein FtsZ (*ftsz*) and protein translocase subunit SecA (*secA*), were selected as candidate reference genes. Their selection was based on the results of previous work. Among these nine genes, *proC* [14], *recA* [15], *rho* [16] and *gyrA* [17] have been selected as stable internal control genes under different conditions in other organisms (*Pseudomonas aeruginosa*, *Lactobacillus casei* and *Staphylococcus aureus*), but have not been studied in *P*. *brassicacearum* GS20. Therefore, for the first time, we quantified the changes in expression of these nine candidate reference genes for *P*. *brassicacearum* GS20 at different growth phases and under various conditions. The stability of expression was measured by Bestkeeper, NormFinder and GeNorm programs. The selected reference genes were then used to validate the expression of the iron-regulating gene from *P*. *brassicacearum* GS20 under different iron conditions.

## Materials and methods

### Selection of candidate reference genes and design of primer pairs

Nine genes (*recA*, *gyrA*, *rpoD*, *gmk*, *rho*, *16S*, *ftsz*, and *secA*) were selected to be used for real-time qRT-PCR-based relative expression analyses of *P*. *brassicacearum* GS20 target genes. These genes have been used as internal reference genes in other studies [14, 15, 18] and are listed in Table 1.

**Table 1:** Candidate reference genes and primers sequences used for real-time-qPCR (Fw = forward primer; Rv = reverse primer; ND = not determined).

Gene names	Gene description	Primer 5’-3’		Amplicon size	R^2^	Efficiencies (%)
*16S*	16S ribosomal RNA	Fw	TTGGGAGGAAGGGCATTAAC	108 bp	0.995	100.67
		Rv	CGCTTGCACCCTCTGTATTA			
*gmk*	Guanylate kinase	Fw	AGTGAACGGCGTGAACTATC	118 bp	0.976	103.06
		Rv	GCTTTGCGAAGTGCCATAAA			
*rpoD*	RNA polymerase sigma factor	Fw	GCCGAGATCAAGGACATCAA	100 bp	0.999	105.50
		Rv	AGATCACCAGACGCAAGTTC			
*ftsZ*	Cell division protein FtsZ	Fw	GCTAGCCCGGTTATCAAAGT	101 bp	0.992	115.36
		Rv	AGTGTTGGCGCAGATGAA			
*secA*	Protein transloca- se subunit SecA	Fw	CCAAGCTGTACATCGAGATCAA	110 bp	0.982	96.79
		Rv	GGTCTTCTCATCAACGGTGTAG			
*gyrA*	DNA gyrase A	Fw	CGAGCTGGTGAAAGAGAAGAA	131 bp	0.999	105.89
		Rv	CGTAGAGGTTGTTGAGGATCAC			
*proC*	Pyrroline-5-carboxylate reductase	Fw	AACACGGCATCGAAACCT	131 bp	ND	ND
		Rv	TTGATGAGGCTTGAGACTTGG			
*recA*	Recombinase A	Fw	GCGGTGAAAGAAGGTGATGA	119 bp	0.999	105.03
		Rv	GTAGATGCCCTTGCCGTAAA			
*rho*	termination factor Rho	Fw	AATACACCGCCGAGCAAA	121 bp	0.990	107.24
		Rv	CCACCGGATTGTTTGTTAACTG			

With reference to the whole genome sequence of *P*. *brassicacearum* GS20 (unpublished), candidate gene primers were designed using the primer analysis software Primer 3 Web Version 4.1.0 (http://bioinfo.ut.ee/primer3/). Details of the primers are provided in Table 1. The effectiveness of the amplification was verified by conventional PCR and the product size was checked by electrophoresis on 3% agarose gels. All of the nine primers amplified single products, as expected (S1 Fig).

### Bacterial cultures and sample processing

Prior to each experiment, *P*. *brassicacearum* GS20 was inoculated from Luria-Broth (LB) agar plates [10 g/L tryptone, 5 g/L yeast extract, 10 g/L NaCl and 1.5% agar (w/v)] and cultured overnight in LB liquid medium (10 g/L tryptone, 5 g/L yeast extract and 10 g/L NaCl, pH 7.0) at 30°C on a rotary incubator shaker (180 rpm). The relative expression of the nine candidate reference genes was analyzed at different growth phases and under various growth conditions.

Growth phases: *P*. *brassicacearum* GS20 was cultured in LB liquid medium at 30°C and pH 7.0; bacterial cells were harvested in the lag phase (OD_600_ = 0.2), exponential phase (OD_600_ = 0.6) and stationary phase (OD_600_ = 2.0). The culture time of these three growth phases was 6h, 12h and 36h respectively.

Temperatures: The strain was cultured overnight in LB liquid medium at three different temperatures (25°C, 30°C and 37°C) and the pH was 7.0. The bacterial cells were collected during the exponential phase (OD_600_ = 0.6).

pH values: The strain was cultured overnight in LB liquid medium under three pH values (pH 5.0, pH 7.0 and pH 9.0) and at 30°C. The bacterial cells were collected during the exponential phase (OD_600_ = 0.6).

Iron levels: The expression stability of the proposed reference genes was analyzed under different concentrations of iron in LB culture medium. The high iron group was cultured in LB containing ferrous iron (FeSO_4_); the low iron group was cultured in LB containing an iron chelator (2,2′-dipyridyl); the control group was cultured in LB containing MnSO_4_, which provides divalent ions (Mn^2+^), for the studies of Fur-mediated iron regulation. The final concentrations of FeSO_4_, 2,2’-dipyridyl and MnSO_4_ were 100 μM each in LB culture media. *P*. *brassicacearum* GS20 was initially cultured for 8 h in LB liquid medium at 30°C and pH 7.0; then, the bacterial cells were harvested 30 min after the addition of FeSO_4_, 2,2’-dipyridyl or MnSO_4_.

All of the abovementioned culture samples were conducted in triplicate. The bacterial cells were centrifuged (at 4°C and 3074 *g* for 10 min) and collected in Eppendorf tubes before extraction of total RNA.

### Total RNA extraction and cDNA synthesis

Total RNA was extracted from 5 mL of fresh culture using TRIzol reagent (Invitrogen, Carlsbad, CA, DNA). The RNA was dissolved in ribonuclease-free water and its purity and concentration were quantified using a NanoDrop spectrophotometer (ND-2000; NanoDrop Technologies Inc., Wilmington, DE, DNA). RNA integrity was analyzed by 3% agarose gel electrophoresis.

Approximately 1 μg RNA was used for real-time PCR in each sample. Complementary DNA (cDNA) was synthesized with a PrimeScript RT reagent kit with genomic DNA (gDNA) Eraser (Perfect Real Time, Takara Biochemicals, Dalian, China), according to the manufacturer’s instructions. The kit eliminated gDNA residues before the synthesis of first-strand complementary DNA by the following reaction: 5 × gDNA Eraser Buffer (2.0 μL), gDNA Eraser (1.0 μL), total RNA (≤ 1 μg), then RNase-free H_2_O was added up to 10 μL; the reaction system was carried out at 42°C for 2 min. The first-strand cDNA was synthesized in a 20 μL volume reaction containing denatured reaction products (10 μL), PrimeScript RT Enzyme Mix I (1.0 μL), 5 × PrimeScript Buffer (4.0 μL), RT Prime Mix (1.0 μL) and RNase-free H_2_O (4.0 μL). The reaction was conducted at 37°C for 15 min and 85°C for 15 s.

### Real-time-qRT-PCR assay

Prior to the real-time-qRT-PCR assays, the synthesized cDNA samples were diluted with RNase-free water. Real-time-qRT-PCR was performed with TB Green^®^ Premix Ex Taq II (Tli RNaseH Plus, Takara Biochemicals, Dalian, China) and conducted on an ABI StepOnePlus Real-Time PCR System (PerkinElmer Applied Biosystems, Foster City, CA, USA). The reaction mixture included 2 μL of diluted cDNA, each primer (final concentration 400 nM), ROX reference dye (final concentration 500 nM) and 2 × TB Green qPCR SuperMix adjusted with nuclease-free water to a final volume of 20 μL. The primer sequences for the nine selected genes are listed in Table 1. The PCR procedure was as follows: 95°C for 30 s, 40 cycles of 95°C for 5 s and 60°C for 33 s. The melting temperature-determining dissociation step was run at 95°C for 15 s and 60 °C for 1 min and 95°C for 15 s at the end of the amplification. The real-time-qRT-PCR reactions were carried out in triplicate for each cDNA sample.

### Reference gene expression stability analyses

The expression stability of the nine selected reference genes was analyzed using three Microsoft Excel-based tools: BestKeeper [19], GeNorm [20] and NormFinder [21]. For GeNorm and NormFinder, the raw quantification cycle (Cq) values needed to be transformed into relative quantification data by the formula 2 ^(−ΔCT)^. ΔCT included all minus the lowest Cq value among all samples for each reference gene. GeNorm evaluates candidate reference genes by the expression stability measurement (M) value. The lowest M value indicates the most stable expression. Generally, genes with M values ≥1.5 are considered not suitable as reference genes [20]. NormFinder ranks the stability values of all candidate reference genes, similarly to GeNorm; the reference gene with the lowest stability value is considered to be the most stable. NormFinder takes intragroup and intergroup expression variations into account to find the stability value for each candidate reference gene and this algorithm can avoid misinterpretations caused by co-regulated genes. BestKeeper assesses the stability of reference gene expression by the coefficient of variation [CV (% Cq)] and the standard deviation (SD) of the Cq values [SD (±Cq)]. BestKeeper is used to analyze the raw Cq values without any transformation. BestKeeper regards the reference gene with the lowest SD and CV-values to be the most stable. Genes with SD ≥1 are considered to be inconsistent by BestKeeper. Moreover, BestKeeper carries out numerous pairwise correlation analyses between each candidate reference gene and calculates the Pearson correlation coefficient (r) and the probability values. This program considers a higher r value to represent greater stability in gene expression.

### Siderophore halo production under different iron levels

In our previous study, *P*. *brassicacearum* GS20 was shown to be strongly antagonistic to pathogenic fungi and to produce extracellular siderophores on Chrome azurol S (CAS) agar plates. CAS agar plates were prepared according to the method described by Schwyn and Neilands [22]. In order to investigate the production of siderophore halos under different concentrations of iron. FeSO_4_, MnSO_4_, and 2,2’-dipyridyl were added separately to CAS agar plates to final concentrations of 100 μM. *P*. *brassicacearum* GS20 was inoculated on these CAS agar plates and cultured at 30°C for 12 h. The halos on the different CAS agar plates were photographed and measured using a microscope (Leica M205C, Buffalo Grove, IL, USA).

### Validation of Fur-regulated gene (*fhu*) expression

We analyzed the genome sequence of *P*. *brassicacearum* GS20 and found the iron-regulated gene (*fhu*). In order to verify the reliability of the proposed reference genes in the relative expression normalization of *fhu* gene under iron-replete and iron-limited conditions, the fold changes of *fhu* were calculated by the 2^-ΔΔct^ method as the description of Livak and Schmittgen [23]. The relative expression results of *fhu* gene were statistically analysed under different iron contents. Turkey tests of one-way analysis of variance (ANOVA) were used determine the significance of any differences (*P* < 0.01).

## Results

### Real-time qRT-PCR amplification efficiency and specificity of candidate reference genes

Based on previous reports on suitable internal control genes for *Pseudomonas* species, nine candidate reference genes were chosen for this study. Gene names and descriptions, primer sequences, PCR efficiencies, and amplicon sizes are listed in Table 1. The gene *proC* should be pointed out because its Cq values were >30 even when undiluted cDNA was used as the amplification template. Furthermore, we changed its primers twice and the results were the same. Therefore, we excluded *proC* from further study. Primers designed for the amplification were specific and the amplicon sizes were as expected (S1 Fig). All dissociation-curves revealed only single peaks, which indicated that there was no formation of primer-dimers or non-specific PCR products (S2 Fig). Amplification efficiency reflects the amplification rate of a template during the PCR reaction and could be determined by the slope of calibration curve. The acceptable range is generally between 90% and 110% [24]. For all of the candidate genes in this study, the PCR efficiencies extended from 96.78% (*secA*) to 107.24% (*rho*), which were in the acceptable range. Moreover, all correlation coefficients (R^2^) (Table 1) were reasonable and verified the quality of the primers designed for the real-time-qRT-PCR analysis.

### Expression of candidate reference genes under different conditions

**Fig 1:**
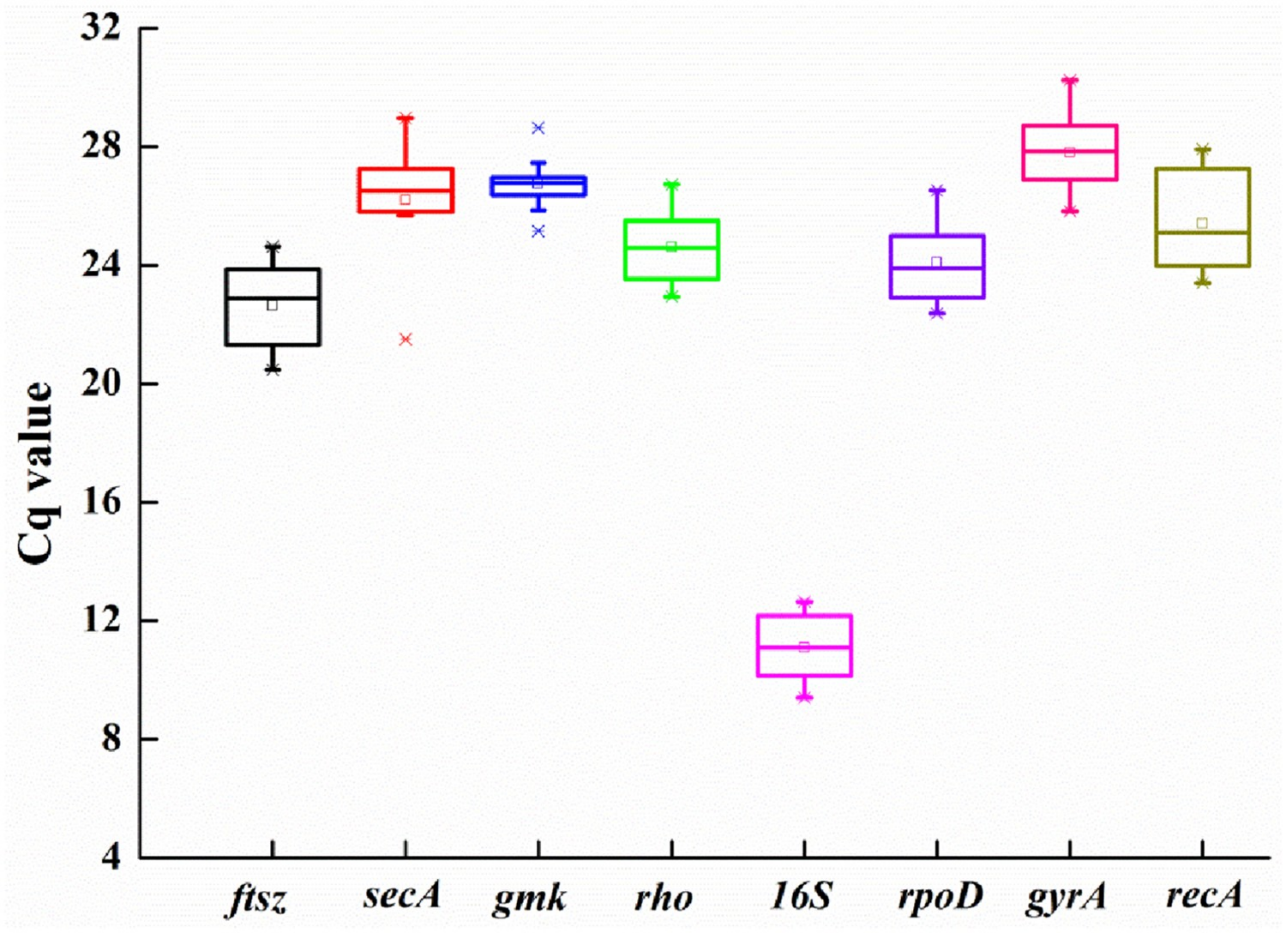
Quantification cycle (Cq) values of eight candidate reference genes.

The candidate reference gene expression levels could be assessed by their Cq values in all samples. As shown in Fig 1, the mean Cq values ranged from 11 (*16S*) to 28 (*gyrA*). Low Cq values designate high expression levels and the opposite indicate low expression levels [25]. The *16S* was the most abundantly expressed gene in all samples (11.27 ± 1.03, mean Cq ± standard deviation), while *gyrA* was the least abundantly expressed gene (28.17 ± 1.35).

### Expression stability analysis by BestKeeper, NormFinder and GeNorm

The expression stabilities of the eight selected candidate genes were evaluated under different conditions and measured by three statistical algorithms: BestKeeper, NormFinder, and GeNorm.

BestKeeper performs repeated pairwise correlation and regression analyses for a given gene with all other genes [26]. The results of the Bestkeeper analysis for the eight genes were shown in Table 2. BestKeeper estimated the standard deviation (SD) of the Cq values [SD (± Cq)] and SD of the absolute regulation coefficients [SD (± x-fold)] and then considered whether a reference gene was acceptable. Generally, suitable reference genes should have values of SD [± Cq] < 1 and SD [± x-fold] <2 [27]. According to this standard, the candidate reference genes *rho*, *16S*, *gyr*, and *gmk* passed this filter and *gmk* had the lowest SD [± Cq] (0.59) and SD [± x-fold] (1.50), while *ftsz* had both the highest SD [± Cq] (1.31) and SD [± x-fold] (2.49). Among the genes *gmk*, *16S*, *gyrA* and *rho*, *16S* exhibited the highest coefficient of variation (CV) value (7.77). BestKeeper performed pairwise comparisons and calculated coefficients of correlation (r). The r value is related to the stability of gene expression and the ideal r value is 1, which indicates the highest gene expression stability. Among *rho*, *16S*, *gyrA* and *gmk*, the r value of *rho* (0.924) was the closest to 1, so it was the most stable gene. The *gmk* was the least stable gene and its r value was 0.783.

**Table 2:** Analysis of eight candidate reference genes using the Bestkeeper algorithm.

Gene name	gmk	16S	gyrA	rho	rpoD	secA	recA	ftsz
Geo Mean [Cq]	26.76	11.03	27.77	24.60	24.06	26.11	25.37	22.60
Min [Cq]	25.21	9.56	25.88	22.97	22.44	21.52	23.42	20.50
Max [Cq]	28.57	12.54	30.06	26.07	26.51	28.80	27.88	24.59
SD [±Cq]	**0.59**	**0.86**	**0.92**	**0.96**	1.13	1.17	1.29	1.31
CV [%Cq]	2.20	7.77	3.31	3.90	4.68	4.48	5.06	5.81
Min [x-fold]	-2.93	-2.77	-3.72	-3.10	-3.06	-24.07	-3.85	-4.28
Max [x-fold]	3.51	2.85	4.89	4.29	5.46	6.48	5.72	3.98
SD [±x-fold]	1.50	1.82	1.89	1.95	2.18	2.25	2.44	2.49
Coeff. of Corr. [r]	0.783	0.866	0.832	**0.924**	0.929	0.463	0.407	0.836
*p*-value	0.007	0.001	0.003	0.001	0.001	0.177	0.243	0.003

[Cq] = cycle quantification; Geo Mean [Cq] = geometric mean of Cq; Min and Max [Cq] = the extreme values of Cq; SD [Cq] = standard deviation of Cq; CV [%Cq] = coefficient of variance of Cq and expressed as percentage; Min [x-fold] and Max [x-fold] = the extreme values of expression levels presented as an absolute x-fold over- or under-regulation coefficient; SD [± x-fold] = standard deviation of the absolute regulation coefficients, Coeff. of Corr.[r] = coefficient of correlation between each candidate and the BestKeeper index.

NormFinder also measures candidate gene stability. Based on this algorithm, the most stable candidate genes were usually those with the lowest stability values. The stability values of the eight genes in all samples are shown in Table 3. Under different pH values, *ftsz* (0.062) was the most stable gene, while *recA* (0.651) was the least stable. The *ftsz* (0.318) was ranked highest, whereas *gmk* (0.087) was the most stably expressed under different temperatures. At different growth phases, *rho* was the most stable gene with an M value of 0.024 and *recA* was the least stable gene with an M value of 0.78. Under different iron levels, the M values of all eight candidate reference genes were lower than other samples and *gyrA* (0.012) was the most stable, while *secA* (0.086) was the least stable. When all these different conditions were taken into consideration, the stability values were ranked as *rho* (0.037), *gmk* (0.04), *gyrA* (0.06), *rpoD* (0.065), *ftsz* (0.08), *16S* (0.095), *secA* (0.098) and *recA* (0.256). Therefore, according to these analyses, *rho* was the most stable gene, which was in accordance with the BestKeeper result, and *recA* was the least stable. Moreover, *gmk* and *rho* gave the best combination with a stability value of 0.028.

**Table 3:** Eight reference genes ranked by their expression stability calculated by NormFinder.

Ranking	Total		pH values		Temperatures		Growth phases		Iron levels	
	Gene	Stability Value	Gene	Stability Value	Gene	Stability Value	Gene	Stability Value	Gene	Stability Value
1	*rho*	0.037	*ftsz*	0.062	*gmk*	0.087	*rho*	0.024	*gyrA*	0.012
2	*gmk*	0.040	*rho*	0.070	*rpoD*	0.100	*gmk*	0.029	*16S*	0.019
3	*gyrA*	0.060	*16S*	0.083	*gyrA*	0.112	*ftsz*	0.137	*rpoD*	0.020
4	*rpoD*	0.065	*gmk*	0.097	*secA*	0.126	*gyrA*	0.169	*rho*	0.023
5	*ftsz*	0.080	*gyrA*	0.122	*rho*	0.140	*rpoD*	0.206	*recA*	0.032
6	*16S*	0.095	*rpoD*	0.128	*16S*	0.286	*16S*	0.268	*ftsz*	0.034
7	*secA*	0.098	*secA*	0.171	*recA*	0.305	*secA*	0.294	*gmk*	0.064
8	*recA*	0.256	*recA*	0.651	*ftsz*	0.318	*recA*	0.782	*secA*	0.086

**Fig 2:**
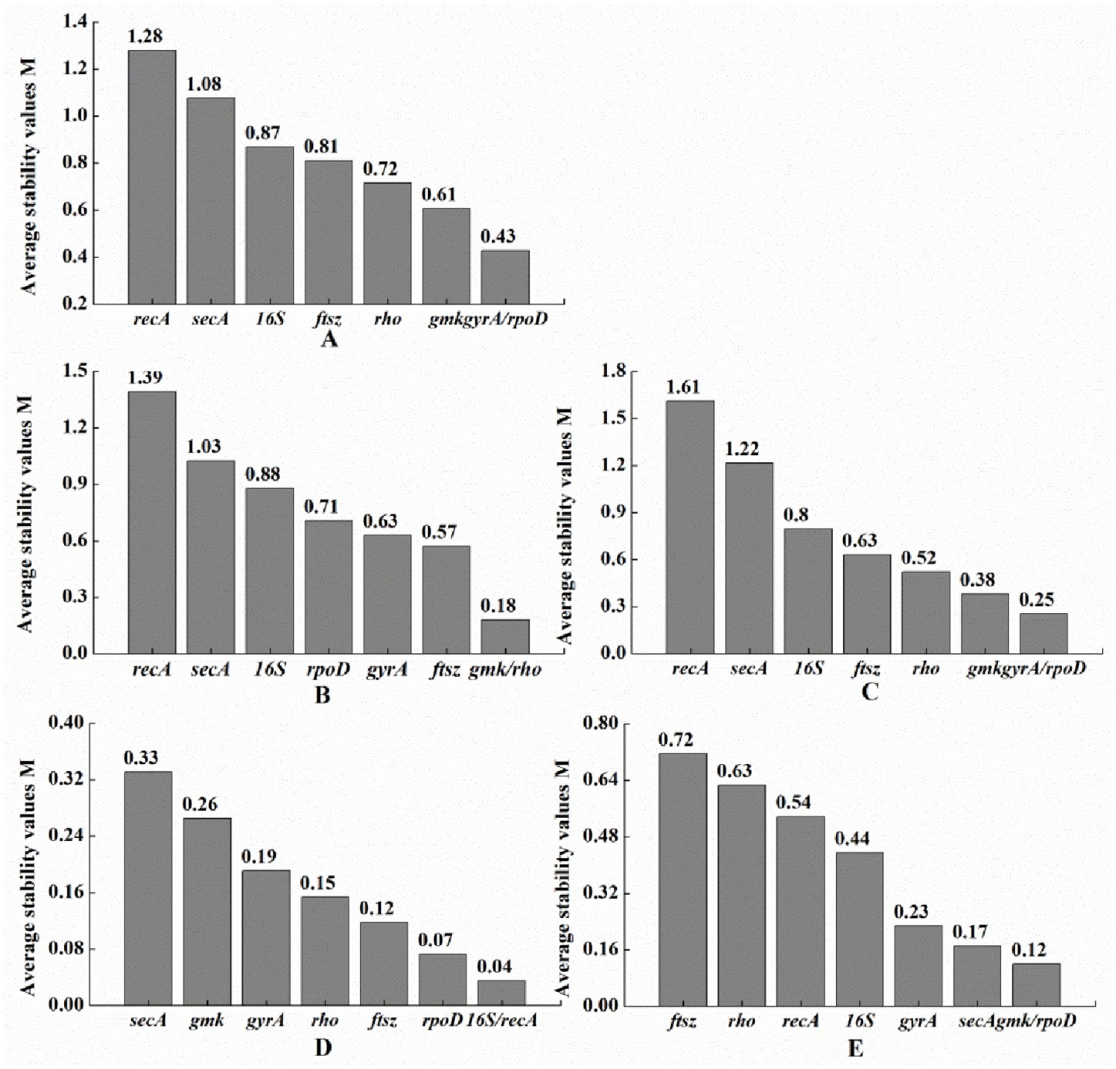
The expression stability of eight candidate reference genes in all samples as determined using GeNorm.

Finally, GeNorm was also used to investigate gene expression stability in terms of M values. The most stable reference genes have the lowest M values. All eight candidate genes had M values lower than the proposed cutoff value of 1.5 under most conditions (Fig 2). For different growth phases, *gmk* and *rho* performed the best with the lowest M value of 0.18, while *recA* had the highest M value of 1.39. *gyrA* and *rpoD* were the most stable reference genes with the M value of 0.25, but *recA* was the least stable with an M value of 1.61 under different pH conditions. At different temperatures, *gmk* and *rpoD* exhibited the most stability with the M value of 0.12, whereas *ftsz* was the most unstable gene with an M value of 0.72. Similarly to the results obtained using NormFinder, the M values under different concentrations of iron were lower than for the other conditions. The M values of *16S* and *recA* were the lowest (0.04), while the M value of *secA* was the highest (0.33). In analyzing all of the conditions together, *gyrA* and *rpoD* ranked as the most stable genes, with an M value of 0.43, while *recA* had the highest value for M at 1.28 and, therefore, showed the least stable expression under all conditions.

**Fig 3:**
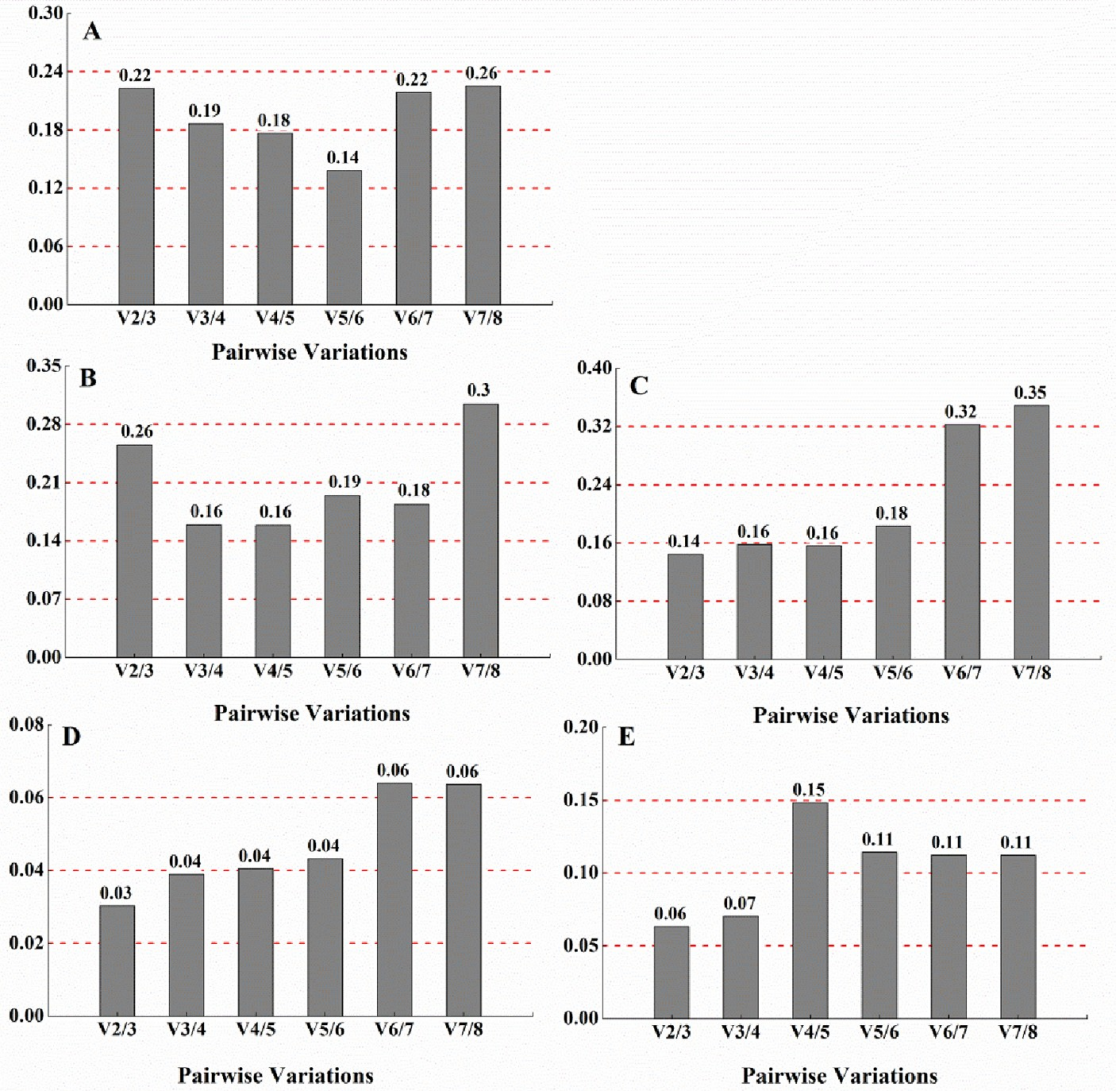
The pairwise variation values of eight reference genes obtained using GeNorm analysis.

GeNorm was also used to determine a suitable number of reference genes required for the precise normalization of target gene expression by pairwise variation (V value). The V values of the eight reference genes under different conditions are shown in Fig 3. For different pH values, temperatures, and iron levels, the V_2/3_ value was lower than 0.15, which was below the proposed V value threshold of 0.15. This meant that the first two genes were enough to be used for the validation. At different grow phases, all the V values were more than 0.15. In analyzing all of the different conditions together, the V_5/6_ value was 0.14; hence, the first five genes (*gyrA*, *rpoD*, *gmk*, *rho*, and *ftsz*) were enough to provide accurate validation and there was no need to introduce *16S* to improve it. In fact, the V value is only used to guide the selection of the optimal number of reference genes and the proposed value (0.15) should not be taken as a strict cut-off [20]. Usually, the three best reference genes are used for a valid normalization strategy, which can result in a more reliable normalization than the use of only one reference gene.

Based on the algorithms of BestKeeper, NormFinder and GeNorm, the expression stability of the eight reference genes was not exactly in accordance. Therefore, we confirmed the rank of the eight reference genes using the geometric mean of the rankings from the three programs (Table 4). The result showed that *rho* was the most stable gene, followed by *gyrA* and *rpoD* and, finally, *recA*. Therefore, *rho*, *gyrA*, and *rpoD* were selected as the most stable internal reference genes for accurate validation in the subsequent study.

**Table 4:** The stability ranking of eight candidate genes by geNorm, NormFinder and BestKeeper (ND = not determined).

Ranking	GeNorm	NormFinder	BestKeeper	Geometric mean of rankings	
1	*gyrA*	*rho*	*rho*	*rho*	1.59
2	*rpoD*	*gmk*	*16S*	*gyrA*	2.08
3	*gmk*	*gyrA*	*gyrA*	*rpoD*	2.82
4	*rho*	*rpoD*	*gmk*	*gmk*	2.88
5	*ftsZ*	*ftsZ*	ND	*ftsZ*	5
6	*16S*	*16S*	ND	*16S*	6
7	*secA*	*secA*	ND	*secA*	7
8	*recA*	*recA*	ND	*recA*	8

### Validation of Fur-regulated gene variation under different iron levels

**Fig 4:**
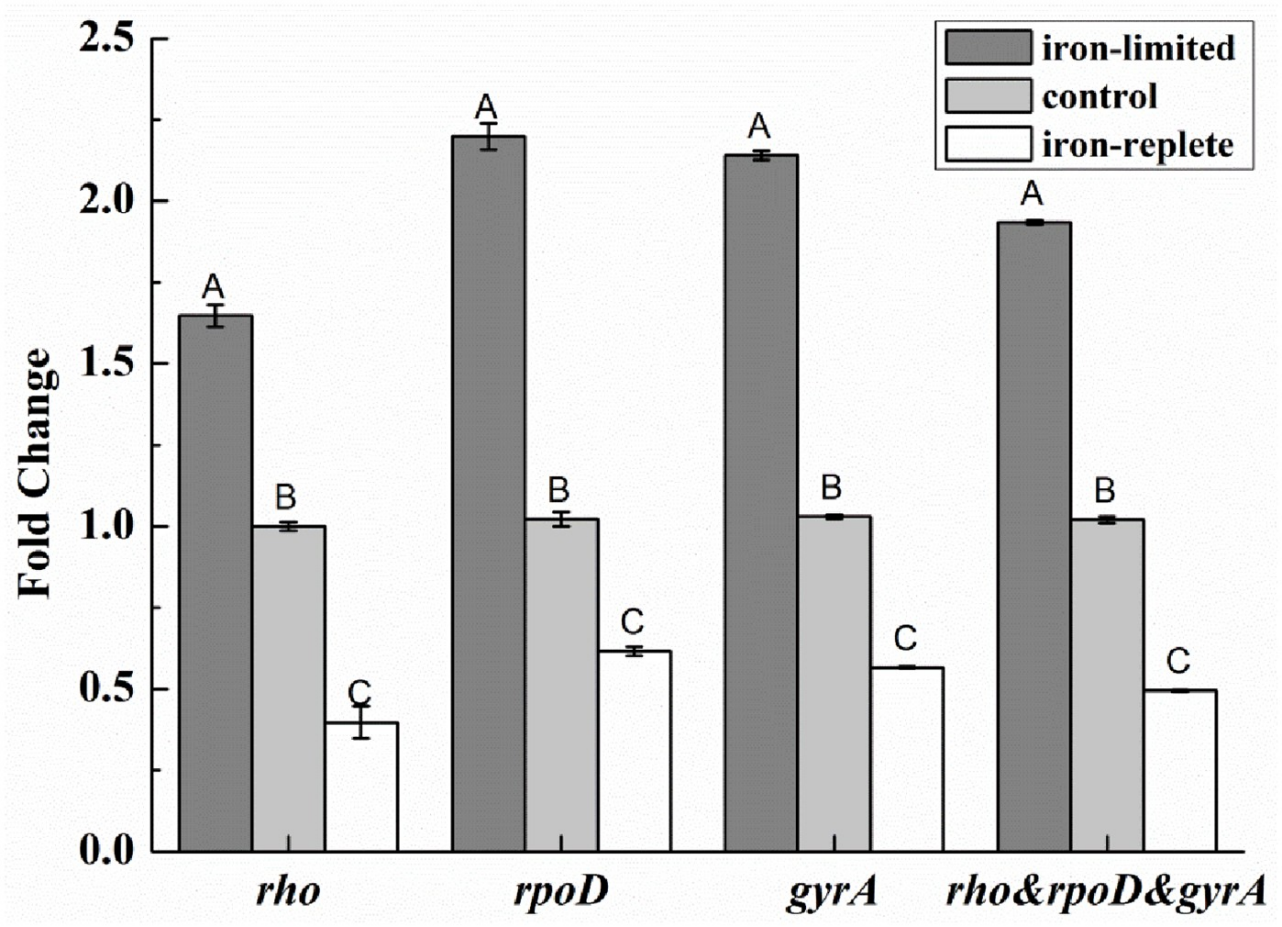
Relative expression of *fhu* gene validated by different reference genes.

**Fig 5:**
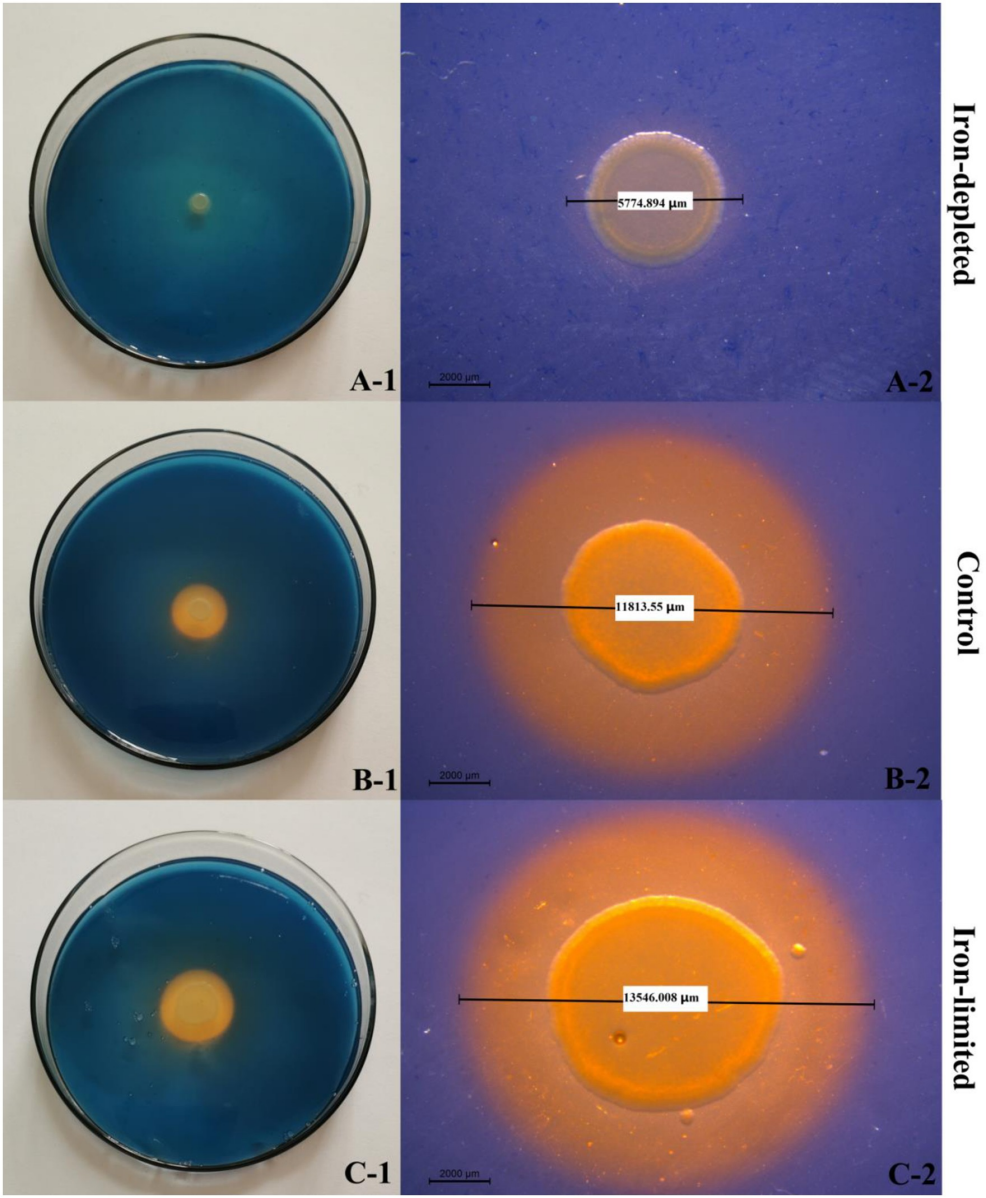
The production of siderophores on CAS plates with different levels of free iron.

The genes *rho*, *gyrA* and *rpoD* were selected as the most stable reference genes to validate Fur-regulated gene variation under iron-replete and -limited conditions. As shown in Fig 4, *fhu* expression was normalized by *rho*, *gyrA*, *rpoD* and their geometric means, respectively; the results of the four kinds of normalization were consistent. They all showed that, when compared with control group, *fhu* was significantly upregulated (*P* < 0.01), and its expression was increased 1.64–2.20-fold in the iron-limited group; while *fhu* was significantly downregulated (*P* < 0.01), and its expression was decreased 0.40–0.61-fold in the iron-replete group. The *fhu* expression differences were in accordance with the variations in siderophore yields under exposure to different levels of iron. We inoculated *P*. *brassicacearum* GS20 onto CAS plates containing different levels of iron; an orange halo zone surrounding the colonies signified the production of siderophores (Fig 5). The diameter of the halo zone was much smaller on the iron-replete plates (5,774.894 μm) than the iron-limited plates (13,546.008 μm).

## Discussion

Quantification of gene expression contributes to the study of cellular responses to changes in conditions and provides an understanding of regulatory mechanisms. To obtain reliable gene expression results using real-time qRT-PCR, stable internal control genes should be selected and used for the accurate normalization of target gene expression [25].

In our study, *P*. *brassicacearum* GS20 had recently been isolated and showed strong antifungal properties, which highlighted its potential as a biocontrol strain. There are no previous reports of validated reference genes for *P*. *brassicacearum*. There were several studies relating to internal reference genes for *Pseudomonas* species, but the results were inconsistent. Savli *et al*. (2003) studied the expression stability of six housekeeping genes (*ampC*, *fabD*, *proC*, *pbp-2*, *rpoD*, and *rpoS*) in *P*. *aeruginosa* and found that *proC* and *rpoD* comprised the most stable pair [14]. Chang *et al*. (2009) investigated the expression stability of eight reference genes (*rpoN*, *rpoD*, *dbhA*, *phaF*, *16S*, *gst*, *lexA*, and *atkA*) in *P*. *putida* mt-2 during its degradation of p-xylene and revealed that *rpoN*, *rpoD*, *16S*, and *atkA* were suitable reference genes with highly stable expression [18]. Alqarni *et al*. (2013) analyzed the expression stability of 13 housekeeping genes (*rpoS*, *proC*, *recA*, *rpsL*, *rho*, *oprL*, *anr*, *tipA*, *nadB*, *fabD*, *ampC*, *algD*, and *gyrA*) under carbon starvation of *P*. *aeruginosa* and found *rpoS* to be the only stably expressed gene [28]. Inconsistencies in reported reference genes attributed to the lack of a universal reference gene for use in gene expression studies and, therefore, the expression stability of reference genes could vary under different experimental conditions [20]. Ideally, the expression stability of each candidate reference gene should be verified under each condition [29]. Other studies used 16S rRNA genes as internal references for validation [30, 31], but transcript levels of *16S* may depend on the state of the bacteria [32]. Also, rRNA genes are abundant and this could affect the accurate quantification of rare and less abundant mRNA transcripts [33]. Moreover, due to their high abundance, the cDNA used needs to be more dilute prior to real-time qRT-PCR reactions; thus, dilution errors may compromise the accuracy of the analyses. Therefore, it was essential to identify suitable internal reference genes for *P*. *brassicacearum* GS20.

In this study, we selected nine candidate reference genes (*recA*, *gyrA*, *rpoD*, *proC*, *gmk*, *rho*, *16S*, *ftsz* and *secA*) to assess their expression stabilities. The programs GeNorm, NormFinder, and BestKeeper were used to analyze data from all the samples. The gene *proC* was excluded due to its low expression quantity, which was different to the result obtained by Savli *et al* [14]. This may be due to *proC* expression differences in the two strains. Based on our analysis of the results calculated by these three programs, we found that the most stable gene was not identical in all conditions but the least stable gene was similar in some conditions. For example, *secA* and *recA* performed as the least stable genes in most conditions (different growth phases, pH values and iron levels), which was in accordance with other studies [34, 35]. So, if the tested samples are from very different treatments, it is important to choose the specific reference gene for validation under each condition [36]. Among the eight genes, the most stable gene was *rho*, as analyzed by NormFinder and BestKeeper, while GeNorm determined *gyrA* and *rpoD* to be the most stable. The pairwise variation determined using GeNorm showed that the top five reference genes (*gyrA*, *rpoD*, *rho*, *gmk* and *ftsz*) could be used as the minimum number required to carry out an accurate normalization of the real-time qRT-PCR data. Based on the different algorithms, the results obtained using GeNorm, NormFinder, and BestKeeper yielded slight differences. Others have reported gene stability ranking differences among these three algorithms [35]. Therefore, taking the three algorithms into consideration, we confirmed the ranking of the eight reference genes’ expression stabilities by calculating the geometric mean of the individual rankings from each of the different algorithms. The result suggested that *rho* ranked first, followed by *gyrA*, *rpoD*, *gmk*, *ftsZ*, *16S*, *secA* and *recA*. Using only one reference gene for validation may affect the accuracy, so deferring to the suggestion provided be the GeNorm result, we selected the three most stable genes (*rho*, *rpoD* and *gyrA*) for the use in validation studies.

To verify the reliability of the selected reference genes, we applied them individually and together to normalize the relative expression of iron-related genes under different iron conditions. They could all objectively reflect the changes in *fhu* expression, even though the fold-change values calculated by the selected reference genes showed some minor difference, which may have been caused by their minor stability differences. By analyzing the stability results, we found the results for fold-change were consistent with the results for stability ranking determined by NormFinder. The ranking of gene stability according to NormFinder was *gyrA*, *rpoD*, and *rho*. Iron-limited conditions could upregulate *fhu* gene expression in *P*. *brassicacearum* GS20 and stimulate the strain to produce more siderophores and obtain iron from the surroundings, thus maintaining normal metabolism. The production of siderophores is an important aspect in antagonizing phytopathogenic fungi, especially under iron-limited conditions [13]. Plant growth-promoting fluorescent pseudomonads could be beneficial as they produce extracellular siderophores under iron-limited conditions and thus utilize environmental iron efficiently and reduce the amount of iron available to other microorganisms, including pathogenic fungi, so as to inhibit their growth [37]. Apart from siderophore production, volatile organic compounds released by *P*. *brassicacearum* GS20 may also inhibit the growth of phytopathogenic fungi (unpublished). Therefore, study of the related antagonistic mechanisms is required. The internal reference genes selected in this study will be valuable to further research in this area.

## Supporting information

S1 Fig Single PCR products of expected size seen using 3% agarose gel electrophoresis. (DOC)

S2 Fig The melting curves of eight candidate reference genes. (DOCX)
