# Supplementary figures and images for: Selection and validation of reference genes for gene expression studies in Pseudomonas brassicacearum GS20 using real-time quantitative reverse transcription PCR
Source: PLoS One. 2020 Jan 27;15(1):e0227927. doi: 10.1371/journal.pone.0227927 (PMC6984700; doi:10.1371/journal.pone.0227927)

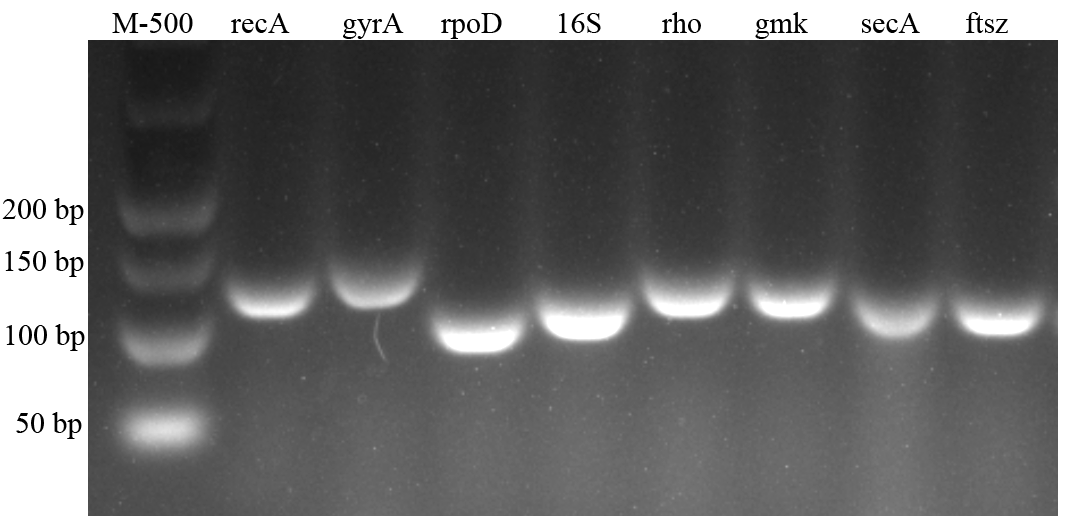


Fig S1. Single PCR products of expected size seen using 3% agarose gel electrophoresis.

Supplement: S1 Fig — (DOC) [file pone.0227927.s001.doc]
