# Supplementary material for: Selection and validation of reference genes for gene expression studies in Pseudomonas brassicacearum GS20 using real-time quantitative reverse transcription PCR
Source: PLoS One. 2020 Jan 27;15(1):e0227927. doi: 10.1371/journal.pone.0227927 (PMC6984700; doi:10.1371/journal.pone.0227927)
Supplement: S2 Fig — (DOCX) [file pone.0227927.s002.docx]

Fig S2. The melting curves of eight candidate reference genes
